# Quantitative assessment of gastrointestinal motility in neonatal rats by real-time 3D photoacoustic imaging: A feasibility study

**DOI:** 10.1016/j.pacs.2026.100853

**Published:** 2026-07-13

**Authors:** Tingting Huang, Li Li, Yu Sun, Yibing Wang, Xinlin Hou, Changhui Li

**Affiliations:** a College of Future Technology, Peking University, Beijing, China; b The Children’s Medical Center, Peking University First Hospital, Beijing, China; c National Biomedical Imaging Center, Peking University, Beijing, China

**Keywords:** Gastrointestinal imaging, Photoacoustic imaging, Intestinal motility, 3D dynamic imaging

## Abstract

Gastrointestinal (GI) motility assessment in neonates remains challenging for conventional clinical imaging. We present a preliminary quantitative framework for GI dynamics in healthy neonatal rats using a real-time three-dimensional photoacoustic imaging system, enabling high-contrast visualization of gastric and intestinal structures. India ink was administered orally as a contrast agent to enhance visualization of the GI tract. We developed a dual-branch analysis pipeline including spatiotemporal kymography for local rhythmic activity quantification and cluster tracking for transport-pattern classification. In healthy neonatal rats, intestinal rhythmic activity was measured at 9.25 ± 0.96 cycles per minute, while gastric peristaltic waves were identified from kymographs. Tracking of contrast-agent clusters revealed local propulsive and retrograde transport patterns, while segmental-like activity was identified from cluster-splitting events. This feasibility study demonstrates a volumetric GI imaging and analysis method for neonatal GI kinematics, laying a foundation for future larger-scale validation and disease-model studies.

## 1 Introduction

The gastrointestinal (GI) system is a highly complex organ network that is essential not only for nutrient absorption but also for immune defense and metabolic regulation [1], [2]. In neonatal medicine, the assessment of intestinal health is particularly important because newborns, especially preterm infants, are highly vulnerable to severe GI disorders due to the immaturity of their digestive, immune, and neuromuscular systems [3], [4]. Necrotizing enterocolitis (NEC), for example, remains one of the leading causes of morbidity and mortality in neonatal intensive care units (NICUs) [5], [6]. NEC progresses rapidly from inflammation and ischemia to bowel necrosis, often requiring urgent intervention [7], [8]. In addition, functional motility disorders, including meconium ileus, intestinal malrotation, and volvulus, also demand prompt and accurate diagnosis to prevent irreversible intestinal damage [9]. Therefore, there is a critical need for non-invasive bedside imaging techniques that can simultaneously visualize GI structure, monitor dynamic motility, and evaluate intraluminal transport.

However, currently available clinical imaging modalities still have limitations for bedside GI monitoring. Computed tomography (CT) provides high-resolution three dimensional(3D) anatomical information, but the use of ionizing radiation restricts repeated examinations in neonates [10]. Magnetic resonance imaging (MRI) offers excellent soft-tissue contrast without radiation exposure, yet it is limited by relatively low temporal resolution and sensitivity to motion artifacts [11]. Besides, both CT and MRI also require the transfer of neonates from the NICU to dedicated imaging suites, increasing clinical risks. Owing to its safety, portability, and bedside availability, ultrasound (US) is widely used for neonatal intestinal assessment. However, conventional US is often operator-dependent and generally provides two-dimensional (2D) B-scan results in routine clinical use [12]. Other approaches, such as electromagnetic capsule-based systems, can provide valuable information on transit and localization [13], but they are not suitable for neonates.

Photoacoustic imaging (PAI) has emerged as a promising hybrid modality that combines the high optical absorption contrast of light with the deep penetration and spatial resolution of US [14], [15]. In PAI, pulsed light excitation generates ultrasound through thermoelastic expansion, and the detected acoustic signals are reconstructed to form images of optical absorption distributions in tissue [16], [17]. Owing to these characteristics, PAI has shown strong potential for deep-tissue structural and functional imaging [18], [19], [20]. Recent 3D photoacoustic computed tomography (PACT) studies have further demonstrated dynamic, multiparametric, and whole-body volumetric imaging capabilities in small animals, including tumor angiogenesis and pharmacokinetic imaging, multiplane whole-body spectroscopic imaging, and cerebrospinal-fluid/glymphatic transport monitoring [21], [22], [23]. In GI research, PAI has been explored for assessing intestinal oxygenation [24], visualizing basic motility patterns [25], and detecting inflammatory biomarkers [26]. Related GI photoacoustic studies have also evaluated postprandial intestinal hemoglobin signals, ischemia-associated oxygenation changes, and contrast-enhanced transit function [26], [27], [28]. In addition, exogenous contrast agents such as organic dyes and nanoparticles have been investigated to enhance GI visualization [28], [29], [30], [31]. However, most previous studies have focused on static anatomy or 2D observations. Because the intestine is a highly convoluted and folded tubular network, 2D cross-sectional imaging inherently lacks important out-of-plane information. It is especially difficult for 2D imaging to track moving targets within the intestinal lumen. Therefore, real-time 3D PA imaging is desirable for quantitatively assessing the dynamic GI system, which exhibits nonrigid deformation and complex transport behaviors.

This work presents a preliminary feasibility study of a noninvasive real-time 3D imaging approach for the GI tract based on an advanced PAI system. The main objective is to validate whether real-time 3D PAI can visualize GI dynamic motion in neonatal rats and support extraction of motility-related metrics from volumetric time series. India ink was administered orally to enhance luminal contrast and facilitate motion tracking. Based on high-resolution and high-contrast dynamic 3D GI imaging, we developed a quantitative analysis method to study local GI rhythmic activity and classify intestinal transport patterns. The present study was performed in healthy neonatal rats. Future disease-model studies will be required to evaluate its utility for conditions such as motility cessation, abnormal transit, segmental dysmotility, delayed gastric emptying, and NEC-related intestinal dysfunction.

## 2 Methods

### 2.1 Real-time 3D PA imaging system

**Fig. 1:**
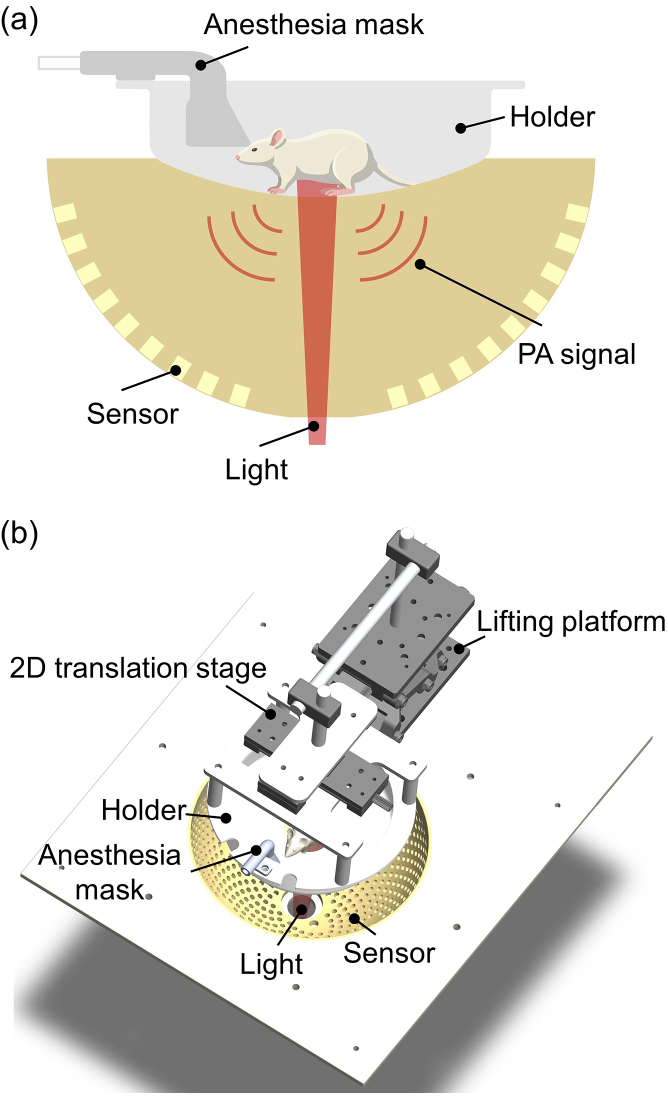
Schematic illustration of the 3D PAI system. (a) 2D schematic diagram of the imaging system; (b) 3D structure of the imaging system.

A schematic diagram of the PA 3D imaging system used for neonatal rat intestinal imaging is shown in Fig. 1. The core of the PA imaging platform is a custom-designed hemispherical ultrasonic transducer array with 1024 elements uniformly distributed on its surface(ULSO TECH Inc, China), which has a radius of R = 95 mm [32]. Each element in the array operates at a central frequency of $f_{c}=5$ MHz with a −6 dB fractional bandwidth of approximately 60%. The spatial resolution of the system was characterized using a point absorber formed by placing a tiny black glue drop on the end face of an optical fiber with a 50μm core diameter. For a point target near the center of the hemispherical array, the full width at half maximum was 0.197 mm, 0.200 mm, and 0.196 mm along the X, Y, and Z directions, respectively, indicating approximately 200μm near-isotropic spatial resolution (Supplementary Fig. S1). A 125-point scan with 2 mm spacing around the center further confirmed the spatial-position calibration and resolving capability within the imaging region (Supplementary Fig. S2). This system provides a field of view of $20\times 20\times 10{\text{mm}}^{3}$. For PA excitation, there is an optical window located at the bottom of the hemispherical array, allowing bottom-up illumination. The PA signal is recorded by four 256-channel data acquisition (DAQ) units (Marsonics DAQ, Tsingpai Tech Co., China) working in parallel at a sampling rate of 40 MHz. The hemispherical array is filled with deionized water that is maintained at 37 °C.

The excitation laser source is a tunable nanosecond pulsed laser (SpitLight 600 OPO-10, INNOLAS, Germany), working at a pulse repetition rate of 10 Hz and a width of approximately 6–8 ns. For the experiments described herein, the excitation wavelength was tuned to 750 nm. The free-space laser beam is directed by mirrors and a diverging lens before illuminating the target via the optical access port located at the bottom of the hemispherical array, as indicated by the red beam in Fig. 1(a). The laser beam is expanded to a diameter of approximately 20 mm on the animal surface, resulting in a surface fluence less than $20{\text{mJ/cm}}^{2}$, which is below the American National Standards Institute (ANSI) safety limit of $25{\text{mJ/cm}}^{2}$ for skin exposure at this wavelength [33], ensuring the procedure remains non-damaging.

To facilitate continuous imaging of neonatal rat and improve convenience during the procedure, a customized 3D-printed animal holder was designed specifically for this study. The holder features a circular aperture (4 cm in diameter) at its base sealed with a thin transparent PVC film to allow passage of both laser and ultrasound. The holder is mounted on an xyz translation stage to adjust the position along three axes, as in Fig. 1(b). During imaging, a layer of ultrasound coupling gel is applied onto the PVC film, after which the neonatal rat is positioned directly on the gel layer with its nose covered by an anesthesia mask. We employed a Delay-and-Sum (DAS) reconstruction algorithm accelerated by a high-performance Graphics Processing Unit (GPU) [34]. On the GPU, the reconstruction speed of the system can be matched to the 10 Hz pulse repetition rate of the laser. This enables the operator to visualize, with negligible latency, the reconstructed 2D slices in three orthogonal orientations in real time, thereby providing immediate feedback during the experiment.

### 2.2 Temporal maximum value projection for gastrointestinal lumen visualization

Because the intraluminal contrast agent was transported as spatially discontinuous clusters during peristalsis, a single volumetric frame often showed only partial and fragmented gastrointestinal structures. This clustered distribution is physiologically plausible because intestinal contents are not transported as a uniform continuous column; rather, their spatiotemporal distribution is shaped by propulsive peristalsis, segmental contractions, local mixing movements, and short-range retrograde fluctuations [35], [36], [37]. Segmental motor activity can partition luminal contents into local boluses for mixing, while propulsive waves and local contractions can drive, stretch, split, or temporarily aggregate these contents. To improve visualization of the lumen traversed by the contrast agent over time, we applied a temporal maximum value projection (tMVP) method to the temporal 3D PA sequence. For a time-resolved PA volume sequence I(x,y,z,t), the tMVP volume was defined as:

(1) $$I_{\mathrm{tMVP}}\left(x,y,z\right)=\underset{t\in \left[t_{1},t_{2}\right]}{\max}I\left(x,y,z,t\right),$$

where $\left[t_{1},t_{2}\right]$ denotes the selected observation window. This operation assigns each voxel the maximum PA amplitude observed during the selected period, thereby integrating the spatial locations visited by the contrast agent into a single volumetric representation. The tMVP image was used only for visualization of the cumulative contrast-agent pathway and anatomical continuity of the GI lumen, whereas quantitative motility analysis, including kymography and 3D centroid tracking, was performed on the original time-resolved volumetric data.

### 2.3 Framework of quantitative motility analysis

**Fig. 2:**
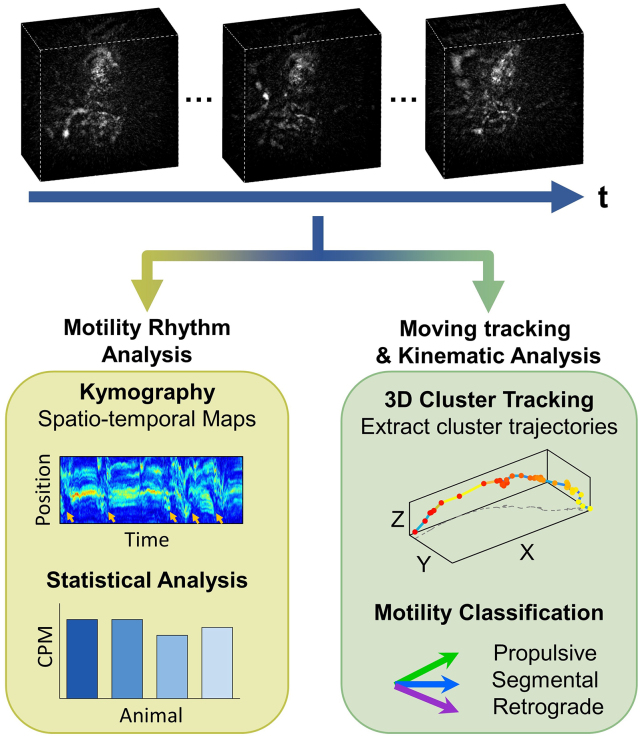
GI motility analysis framework based on dynamic 3D PAI. Temporal 3D volumetric images are analyzed through two pipelines: (left) motility rhythm analysis using kymography to generate spatiotemporal maps and contraction motion; and (right) kinematic and functional analysis involving 3D cluster tracking for trajectory extraction and classification of propulsive and retrograde transport, with segmental-like activity identified from cluster-splitting events.

Based on real-time 3D imaging results acquired by PAI, we proposed the framework for quantitative analysis of GI motility, as illustrated in Fig. 2. In principle, the acquired temporal 3D volumetric serial images are processed through two analysis pipelines. In the left pipeline, the motility rhythm is quantified. Specifically, kymography is utilized to generate spatiotemporal maps, from which motion pattern is examined to identify the contraction event and count its frequency, expressed as cycles per minute (CPM). Line segments used for kymography were manually selected according to predefined criteria: the segment should pass through a GI region with clear PA signal and visible motion, be placed as closely as possible along the local GI lumen or the main direction of content motion, and maintain stable signal visibility during the 60 s observation window. For gastric analysis, the line was selected in a high-contrast ink region where peak displacement associated with peristaltic waves was visible. For intestinal analysis, the line was selected in a local intestinal segment containing a high-signal region with periodic reciprocal or propulsive motion. The right pipeline focuses on motion tracking and kinematic analysis. This is achieved by real-time 3D tracking of contrast agent cluster in intestinal lumen to extract trajectory, which enables the measurement of speed and the classification of motility patterns.

**Fig. 3:**
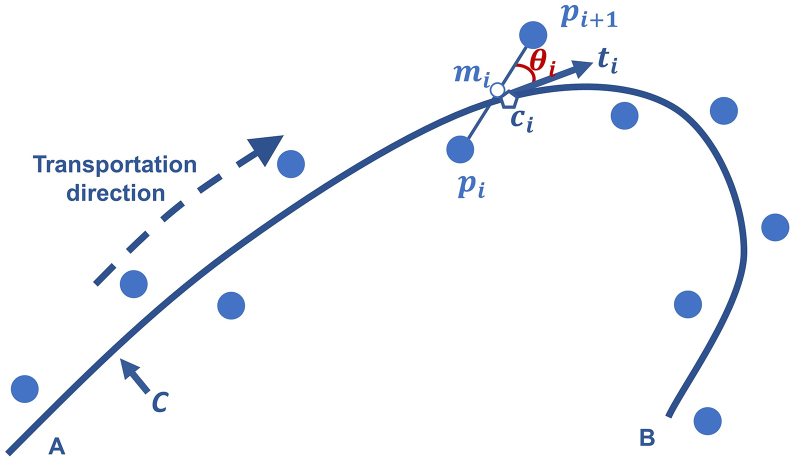
Tracking the target and constructing a smoothed local reference centerline from the true 3D centroid positions of the selected intraluminal cluster.

The contraction rhythm frequency can be manually counted from the corresponding kymography results. The kinematic analysis requires calculating the speed and direction of the targets. Clusters used for 3D centroid tracking were selected when they showed PA signal clearly higher than the background, relatively clear spatial boundaries within the selected time window, sufficient separation from nearby high-absorption structures, and stable identification across consecutive frames. If a cluster split, the leading sub-cluster in the overall migration direction was selected for subsequent tracking; after fusion, the fused cluster was used as the current tracked position. Tracking loss was defined as disappearance of the target signal or exit of the target from the field of view, at which point tracking was terminated. In this feasibility study, single-cluster tracking was used to demonstrate the analysis workflow because the current procedure still requires manual target selection and because multi-cluster statistical analysis will require larger datasets and automated algorithms. As demonstrated in Fig. 3, first, we continuously tracked a specific cluster of the contrast agent over a certain time, obtaining its centroid coordinates $p_{i}=\left(x_{i},y_{i},z_{i}\right)$ at each frame number i. Each $p_{i}$ represented the experimentally measured true centroid in the original time-resolved 3D PA volume. The reference curve C was not an anatomical segmentation of the entire intestinal lumen, but a smoothed local centerline generated from this centroid trajectory. The trajectory was optionally trimmed only for tube construction when a terminal return segment caused artificial tube overlap, smoothed and simplified, and then resampled at uniform arc-length positions using makima interpolation. A circular cross-section was swept along C for tube visualization, whereas the original adjacent-frame centroid displacements were retained for speed and direction analysis.

For each interval between two adjacent frames, the displacement vector of the tracked cluster and its absolute propagation speed are calculated as:

(2) $$r_{i}=p_{i+1}-p_{i}$$

$$v_{i}=\frac{\left|r_{i}\right|}{\Delta t}$$

Then, to determine the peristaltic direction, each adjacent-frame displacement was compared with the local tangent of its nearest point on curve C:

(3) $$m_{i}=\frac{p_{i+1}+p_{i}}{2},$$

$$k^{*}=\arg\underset{k}{\min}{\left\|C_{k}-m_{i}\right\|}_{2}^{2},$$

$$a_{i}=\frac{r_{i}^{⊤}t_{k^{*}}}{{\left\|r_{i}\right\|}_{2}{\left\|t_{k^{*}}\right\|}_{2}},\;\theta_{i}=\arccos\left(a_{i}\right).$$

Segments with $\theta_{i}\leq 90{}^{\circ}$ were classified as propulsive, whereas segments with $\theta_{i}>90{}^{\circ}$ were classified as retrograde. Segments with non-positive time intervals or negligible displacement were excluded from this angle-based calculation. The propulsive and retrograde classification in this study should be interpreted as forward and backward motion relative to the local overall migration direction of the selected cluster, rather than as an anatomically validated global oral-to-anal direction.

### 2.4 Animal preparation

The study utilized healthy neonatal Sprague-Dawley rats at postnatal days 2–3, with body weights ranging from 7.5 to 9.5 g. All animal procedures were conducted in strict compliance with the ethical guidelines and protocols approved by the Animal Ethics Committee of Peking University First Hospital (Approval No. J2023048). Contrast-free imaging was performed 2–3 h after feeding. The rats were placed on an animal holder in a prone position with the abdomen facing the hemisphere array. Anesthesia was maintained with 1.5% isoflurane in air at a flow rate of 1 L/min throughout the imaging experiment. A total of four neonatal rats were imaged. All four animals underwent baseline and post-gavage imaging. Late-digestion follow-up imaging at 24 h was performed in one animal as a feasibility demonstration.

### 2.5 Contrast agent administration

In order to specifically enhance the visualization of the gastrointestinal lumen, in this study, we used India ink (S30881, YuanYe Bio, China) as the exogenous contrast agent. India ink is primarily composed of carbon particles with high optical absorption and has been commonly used as a high-absorption intraluminal tracer in preclinical GI imaging studies. Moreover, it is expected to remain primarily within the gastrointestinal lumen, making it suitable for tracking intraluminal transport. The ink was diluted at a ratio of 1:5 with deionized water. A total volume of 100 μL of the diluted contrast agent was administered via oral gavage to the neonates using a precise gavage technique with a No. 6 stainless-steel curved feeding needle measuring 42 mm in length and 0.6 mm in diameter.

## 3 Results

### 3.1 3D PA in vivo intestinal imaging without contrast agent

**Fig. 4:**
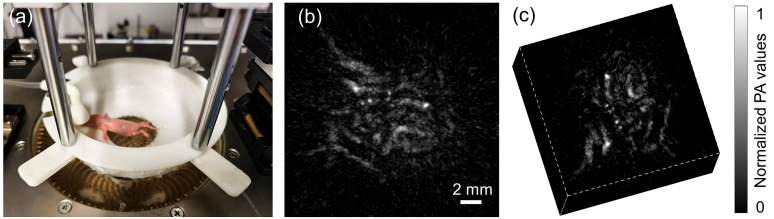
In vivo PAI of the neonatal rat intestine. (a) Photograph of the neonatal rat positioned on the custom holder in the 3D PAI system. (b) MAP reconstruction of the abdominal region. (c) A snapshot of 3D reconstruction result.

Digestive residues or fecal material in the intestinal tract can provide endogenous contrast for PAI. Because the neonatal rats used in this study were at postnatal days 2–3, yellowish milk-derived intestinal contents and bile-related pigments may contribute optical absorption at the 750 nm excitation wavelength [38]. We first performed in vivo intestinal imaging without exogenous agents. As illustrated in Fig. 4(a), the neonatal rat was carefully positioned on the PVC membrane, ensuring its abdominal region was centered relative to the transducer array. A volumetric region of 20 × 20 × 10 mm${}^{3}$ was reconstructed, encompassing most of the abdominal GI region. Fig. 4(b) presents the maximum amplitude projection (MAP) of the 3D reconstructed intestine, demonstrating complex structures of the intestine. It was observed that the reconstructed intestinal tract exhibited obvious discontinuity and segmentation. This fragmentation may be attributed to the relatively weak PA signals from the thin, less-vascularized intestinal wall, as well as the presence of highly dispersed digestive residues and potential gas pockets along the gut. Fig. 4(c) is a snapshot of the 3D reconstructed images, whose 3D animation is provided in Supplementary Video 1. These contrast-free signals were useful for demonstrating endogenous intestinal PA contrast, but their distribution was sparse and heterogeneous, and the stomach and proximal small-intestinal lumen could not be reliably visualized without exogenous contrast. A representative contrast-free cluster-tracking analysis further showed only limited local displacement and lower speed than the exogenous contrast-agent clusters analyzed in the main results (Supplementary Fig. S3).

**Fig. 5:**
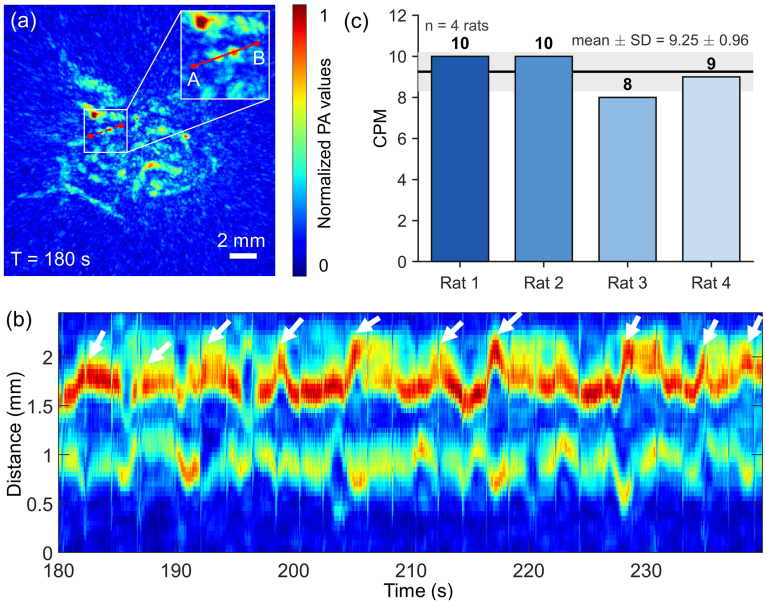
Quantitative analysis of intestinal motility rhythm using 3D PAI. (a) Representative MAP image of the rat abdomen. The red line (A to B) indicates the selected line segment for motility analysis. (b) Spatial–temporal map generated along the selected segment over a 60 s interval (from 180 s to 240 s). The white arrows indicate the peristalsis of the intestinal contents. (c) Statistical analysis of CPM across n=4 rats. Individual CPM values for each rat are labeled above the bars, with an average motility rate of 9.25±0.96 CPM (mean ± SD, indicated by the horizontal black line and shaded area).

The intestinal motility rhythm was quantified through a multi-step image processing workflow, as summarized in Fig. 5. We continuously recorded dynamic intestinal images at a frame rate of 10 Hz for 360 s (a total of 3600 frames). Then, based on acquired frames, a representative segment of the intestine was selected, as marked in Fig. 5(a), which contains a high-contrast cluster that keeps swinging, a sign of intestinal peristaltic motion. We studied a 60 s peristaltic process between 180–240 s, as demonstrated in Supplementary Video 2. The signal intensity along the selected segment, as marked by the red line from A to B in the zoomed-in box of Fig. 5(a) was extracted from each frame over this 60 s period to generate the spatiotemporal kymography, as shown in Fig. 5(b). According to the kymograph, we identified 10 reciprocating motions, highlighted by white arrows, leading to 10 CPM. A total of four animals were studied, yielding a mean local motility rate of 9.25±0.96 CPM. Because of the limited sample size and manual ROI selection, these values should be interpreted as preliminary feasibility data showing that the imaging method can capture local rhythmic intestinal activity, rather than as a normal physiological range for neonatal rats.

### 3.2 Dynamic imaging of GI tract aided by contrast agent

**Fig. 6:**
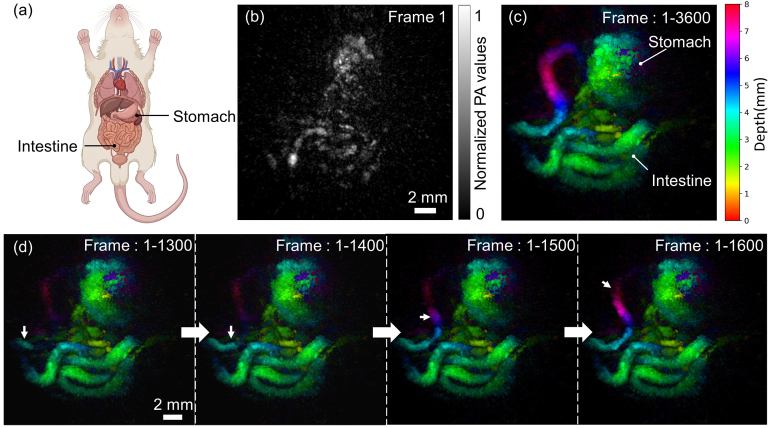
Anatomical illustration and in vivo dynamic PA imaging of the GI tract after oral administration of the contrast agent. (a) Schematic anatomical illustration of the rat, highlighting the stomach and intestine. (b) First frame of PAI MAP. (c) tMVP image generated by taking the maximum PA amplitude over all 3600 consecutively acquired frames collected 10 min after gavage. (d) Cumulative tMVP images reconstructed from frames 1–1300, 1–1400, 1–1500, and 1–1600, respectively. The white arrows indicate the dynamic transit of the contrast agent through a part of intestinal lumen.

To enhance GI imaging quality and enable dynamic tracking of intraluminal transport, we subsequently employed India ink as a GI contrast agent. The exogenous contrast agent was not merely used to increase image brightness; it enabled more complete visualization of the stomach and intestinal lumen, higher signal-to-background ratio in the stomach and small intestine, dynamic transport tracking, and propulsive and retrograde motion classification. After oral gavage administration, the contrast agent gradually transited from the stomach to the intestine. Fig. 6(a) illustrates a sketch of the anatomical diagram including the stomach and the proximal small intestine. We started performing imaging at 10 min post-gavage, as shown in Fig. 6(b). a much clearer stomach image was obtained than was possible without contrast agent in Fig. 4(b) without contrast agent. A portion of ink had already entered the intestinal lumen, contributing to most of the PA signal of the small intestine. Continuous PAI of the GI tract was then performed for 360 s at a frame rate of 10 Hz, the video of which is played at 6× speed in Supplementary Video 3 to provide an intuitive visualization of GI motility.

Although the ink enhanced the PA signal amplitude, Fig. 6(b) also demonstrates that gastric and intestinal motility dispersed fluidic ink solution into small segments, resulting in discontinuous patterns in the reconstructed images. To improve visualization of the cumulative GI lumen occupied by the moving contrast agent, we applied the tMVP method described in Section 2.2. Compared with a single instantaneous frame, the tMVP image integrates all voxels visited by the contrast agent during the selected observation period and therefore provides a more continuous representation of the gastric and intestinal lumen. Fig. 6(c) shows the depth-encoded MAP image generated from the 3D tMVP result using all 3600 frames. Its corresponding 3D animation is provided in Supplementary Video 4. Compared with the single-frame result in Fig. 6(b), the tMVP markedly improves the continuity of the GI tract.

Interestingly, tMVP can illustrate the dynamic propagation of ink driven by intestinal peristalsis. Fig. 6(d) presents depth-encoded tMVP results from frames 1–1300, 1–1400, 1–1500, and 1–1600, respectively. As indicated by the white arrows, it can be observed that the contrast agent progressively advanced along the intestinal lumen, adding new lumen parts in the updated tMVP result.

### 3.3 Gastric peristalsis analysis

**Fig. 7:**
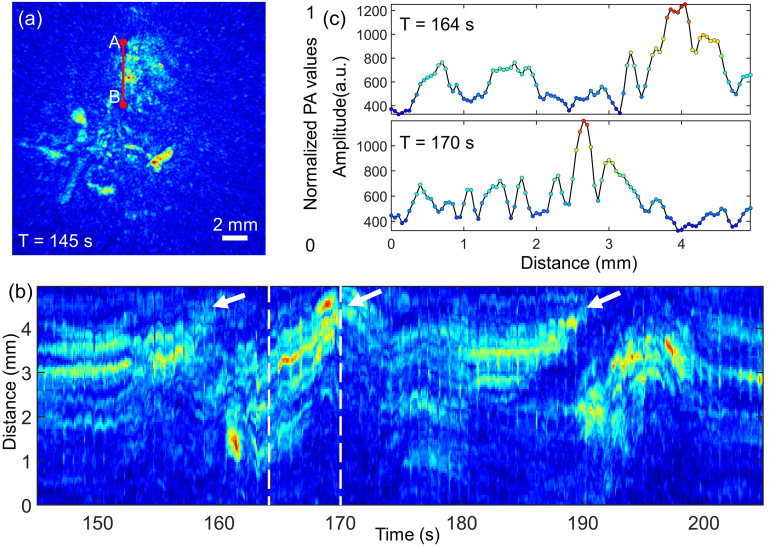
Characterization of gastric peristaltic waves using 3D PAI. (a) Representative MAP image of the rat abdomen. The red line (A to B) indicates the selected line segment along the gastric region for peristalsis analysis. (b) Spatiotemporal kymograph generated along the line segment over a 60 s interval. The pseudo-color intensity represents the normalized PA values. White arrows highlight the gastric peristaltic waves. White dashed lines indicate the specific time points (T=164 s and T=170 s) selected for cross-sectional analysis. (c) Spatial amplitude profiles extracted at T=164 s (top) and T=170 s (bottom). These profiles visualize the signal intensity distribution along the line segment, demonstrating the spatial displacement of the peak PA amplitude (gastric content movement) as the peristaltic wave propagates.

In addition to intestinal peristalsis, when filled with ink, stomach can be imaged with high contrast. Therefore, we can analyze the gastric peristalsis based on the same method used for intestinal peristalsis in Fig. 5. Similarly, we used a line-based kymographic approach applied to the reconstructed result, as illustrated in Fig. 7. A line segment was drawn in the gastric region, as shown in Fig. 7(a). The PA values along this line segment were then extracted frame by frame to construct a spatiotemporal kymograph over a 60 s interval, as presented in Fig. 7(b). Propagating peristaltic events were identified based on the fast changes in pattern in the kymograph. Within this 60 s observation window, three distinct gastric peristaltic waves were identified and marked by white arrows in Fig. 7(b). Fig. 7(c) presents two representative PA values along the line segment at two time points of T = 164 s and T = 170 s, respectively. It can be observed that there is a significant shift in the peak positions of the two profiles, reflecting the movement of gastric contents associated with peristaltic activity.

**Fig. 8:**
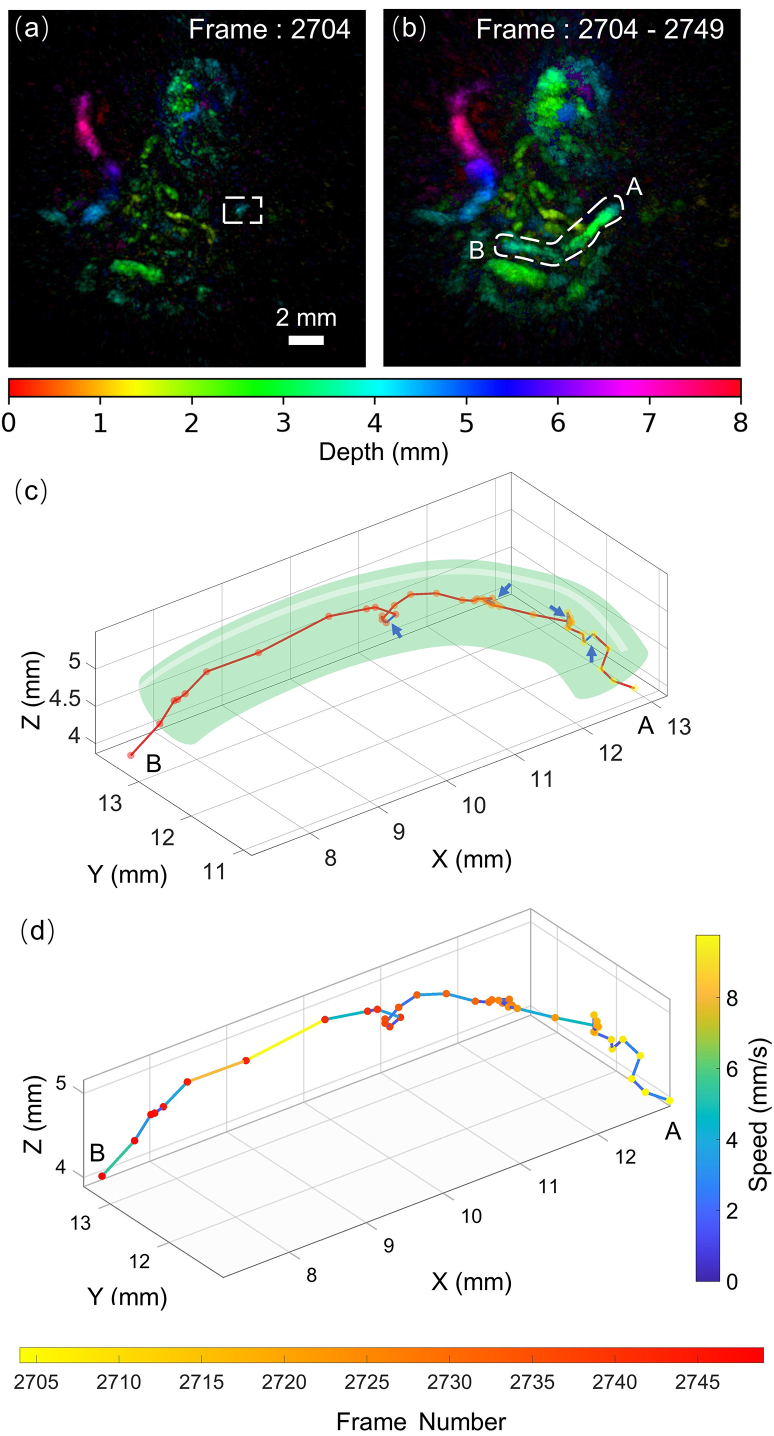
Quantitative analysis of intraluminal contrast agent tracking and motility pattern classification. (a) MAP image of the 3D PA sequence at the initial frame. The white dashed box indicates the tracked cluster for analysis. (b)The right panel shows the tMVP image generated from frames 2704–2749, with the white dashed outline marking the migration pathway of the cluster. (c) Classification of intestinal peristalsis based on moving direction. The semi-transparent green structure represents a schematic virtual intestinal segment. Red markers indicate propulsive peristalsis, while blue markers indicate retrograde peristalsis. Retrograde regions are further highlighted with blue arrows for clarity. Trajectory endpoints are color-coded by frame number to illustrate temporal progression. (d) Visualization of the 3D trajectory of the tracked point. Line segments are colored by speed (mm/s), and scatter points are colored by frame number.

### 3.4 Intestinal 3D kinematic tracking and spatiotemporal motility classification

To study the dynamic characteristics of intestinal motility, 3D tracking was performed to monitor the propagation of the intraluminal contrast agent cluster. As indicated by the white box in Fig. 8(a), a representative cluster satisfying the selection criteria described in Section 2.3 was selected, and the analyzed time window was chosen because the cluster could be continuously identified without tracking loss. Its centroid position was extracted from frames 2704 to 2749 (a period of 4.5 s). A MAP image of the tMVP result from frames 2704 to 2749 was shown in Fig. 8(b), in which the intestinal lumen enclosed by white dashed line delineates the migration pathway of the cluster(from A to B). The dynamic transport of the cluster driven by intestinal peristalsis for this selected segment is shown in Supplementary Video 5.

The motion of the contrast agent was classified using the algorithm described in Section 2.3. Fig. 8(c) depicts the trajectory path of the cluster’s centroid, in which different colors represent different types of motion after classification, including propulsive and retrograde peristalsis. By time, propulsive motion accounted for 75.6% and retrograde motion accounted for 24.4%, presenting an overall forward progression relative to the local reference path of the cluster along the lumen, accompanied by occasional short-range retrograde segments. We calculated the actual propulsive distance to be 8.8 mm and the retrograde distance to be 1.2 mm, corresponding to a propulsive proportion of 87.7% and a retrograde proportion of 12.3%. Fig. 8(d) shows the color-encoded moving speed of the tracked point. The color of each dot indicates the corresponding frame number and the transport speed of the cluster varied substantially. For another time window in the same intestinal lumen (frames 2871 to 2892), the propulsive time and distance proportions were 90.5% and 97.1%, respectively (Supplementary Fig. S4). These results confirm the feasibility of repeatedly extracting local motion direction and velocity information and also indicate that variations occur even within the same segment of the intestine due to differences in digestion time.

**Fig. 9:**
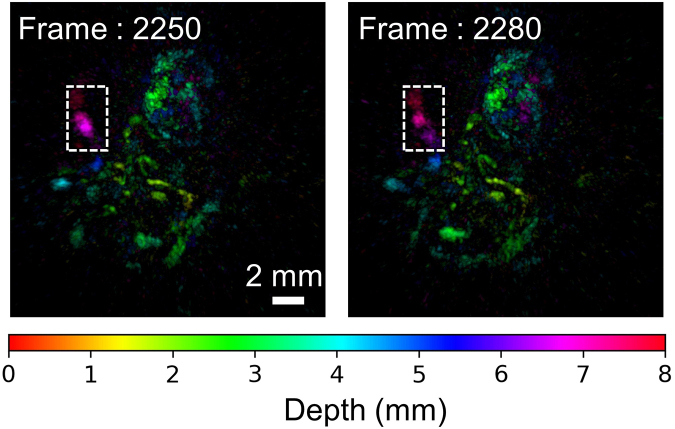
The intestinal segmental motility pattern. A contrast-agent cluster within the white dashed box was split into two sub-clusters over time.

Besides propulsive and retrograde peristalsis, sporadic segmental contractions that split the cluster apart were observed as in Fig. 9, where a cluster in the dashed box was divided into two within 3 s, which can be seen at T = 220 s in Supplementary Video 3. Such splitting, short-range retrograde fluctuations, and local aggregation are consistent with the intrinsic mixing, partitioning, and regulatory functions of intestinal segmental activity [35], [36].

### 3.5 Late-digestion follow-up analysis

**Fig. 10:**
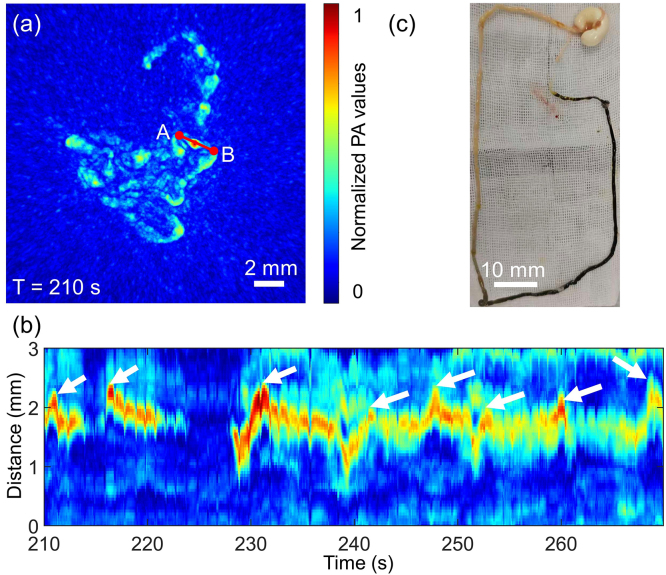
PA imaging and dissection results of the intestine 24 h after contrast agent gavage. (a) Representative MAP result 24 h post-administration of India ink. The red curved line (A to B) indicates the selected longitudinal axis of a functional intestinal segment for motility analysis. (b) Spatial–temporal map generated along the selected segment over a 60 s interval (from 210 s to 270 s). The white arrows indicate the peristalsis of the intestinal contents. (c) Corresponding excised GI tract.

Previous imaging experiments were conducted in the early stage of digestion when the contrast agent was primarily located in the stomach and small intestine. As a late-digestion follow-up demonstration in one animal, 3D intestinal imaging was performed 24 h post-gavage, as shown in Fig. 10(a). The results exhibit markedly improved tubular structural continuity, with the characteristic highly convoluted and folded intestinal architecture well preserved. Compared with Fig. 7, the bulky stomach could hardly be distinguished, indicating complete gastric emptying. At this time point, the PA signals were predominantly localized in the lower abdominal region, such as the cecum and colon. A 3D dynamic animation is provided in Supplementary Video 6, which presents the continuous intestinal dynamics over a 5 s duration. Similarly, we selected a line segment of the intestine and drew the spatiotemporal kymograph covering a 60 s period, as shown in Fig. 10(b). We observed eight peristaltic movements of the intestine (marked by white arrows).

In Supplementary Video 7, we recorded the intestine for 300 s. The video was played at 6× speed, enabling a more intuitive visualization of motility in the cecal and colonic regions. These results confirm the feasibility of real-time 3D dynamic imaging of intestinal motility with the proposed system.

The animal was euthanized immediately after the 24 h imaging experiment, and the photograph of dissected GI tract is shown in Fig. 10(c). Visual inspection of the excised GI tract confirmed that the stomach and proximal small intestine were clear of residual ink, whereas the cecum and large intestine were densely filled with the black contrast agent. Therefore, PA results in Fig. 10(a) do show intestinal structure primarily from the cecum and large intestine.

## 4 Discussion and conclusion

In this feasibility study, we proposed a preliminary quantitative framework to study GI motility in neonatal rats using non-invasive real-time 3D photoacoustic imaging. By combining high-frame-rate volumetric imaging with intraluminal contrast enhancement, we visualized and quantified 3D gastric and intestinal dynamics, including local rhythmic activity and transport patterns. Based on spatiotemporal kymography, we measured an intestinal rhythmic frequency of 9.25±0.96 CPM across four animals and observed gastric peristaltic activity at approximately 3 CPM in a representative recording. The 3D tracking framework enabled classification of local transport direction into propulsive and retrograde components, while segmental-like contractions were identified from cluster-splitting events, demonstrating the potential of 3D tracking for analyzing complex intraluminal motion.

To improve anatomical visualization, we proposed the tMVP algorithm (described in Section 2.2), which successfully restored the continuity of the GI lumen by integrating moving ink clusters. It should be emphasized that while tMVP facilitates structural visualization, the quantitative motility metrics were derived from the original time-resolved data to ensure accuracy.

Several limitations remain. First, the number of animals was limited, and the 24 h follow-up imaging was performed in only one animal. Therefore, the present data are insufficient to establish normal physiological ranges, evaluate interindividual variability, or support strong statistical inference. Second, the current kymography method depends on manually selected line segments. Although moderately shifted line segments in regions with clear PA signals and similar local motion yielded comparable counts in our additional sensitivity analyses, CPM estimation remains sensitive to ROI location, signal quality, out-of-plane motion, and digestive state. Future work should focus on developing automated 3D lumen segmentation and centerline extraction techniques. Third, the 3D cluster tracking analysis was demonstrated mainly with selected single-cluster trajectories. This is appropriate for workflow demonstration, but multi-cluster and cross-animal validation will require larger datasets and automated target-association algorithms. Fourth, the propulsive and retrograde classification is defined relative to a local smoothed migration path, not an independently validated global oral-to-anal anatomical direction. Future studies should combine anatomical localization, fluorescence/white-light video, ultrasound, or other multimodal references to validate absolute transport direction.

Another important limitation is the lack of an independent gold standard for validating PA-derived dynamic 3D motility metrics. A direct reference method would need fast 3D imaging, sufficient spatial resolution for neonatal intestine, non-invasive operation, and high contrast for intraluminal clusters. MRI, PET, optical fluorescence imaging, CT, and ultrafast 3D ultrasound each have potential value but also practical limitations for synchronized validation of spontaneous GI motility. In this study, dissection after 24 h imaging provided anatomical confirmation of contrast-agent distribution, but it did not validate dynamic motility metrics. Future validation may require synchronized multimodal imaging or established GI transit assays adapted to the neonatal rat model.

In addition, while India ink significantly enhanced visualization contrast in this preclinical study, it is a liquid tracer that may not fully replicate the rheological properties of semi-solid chyme. Furthermore, India ink is not a clinically approved agent. Future translational studies must identify clinically acceptable alternatives, such as indocyanine Green (ICG) or modified clinical barium/iodinated suspensions combined with safe near-infrared (NIR) absorbers, as well as food-grade dyes with strong NIR absorption. Besides, future work will include using multi-wavelength spectroscopic PAI, which has potential to monitor parameters like gut wall oxygenation.

Although this study focused on technical and algorithm development in healthy animals, disease-model validation is essential before the practical usefulness of this framework can be established. Future studies should apply this method to pathological animal models, such as neonatal rat models of NEC, and compare healthy and diseased animals under controlled age, body-weight, anesthesia, feeding, and digestive-stage conditions. In NEC and related GI disorders, potentially relevant endpoints may include motility cessation, abnormal transit, segmental dysmotility, delayed gastric emptying, ischemia-related dysfunction, and altered luminal transport. Larger studies with power analysis will be needed to determine whether the proposed metrics can serve as reliable biomarkers.

In conclusion, this study demonstrates the feasibility of real-time volumetric PAI for visualizing and preliminarily quantifying GI motility in neonatal rats. The proposed framework enables measurement of local rhythmic contractions and classification of complex intraluminal transport patterns in 3D. This work provides a methodological basis for future preclinical studies of neonatal gastrointestinal dysmotility and disease-model validation. For clinical translation, a scaled-up hemispherical array with a larger field of view, similar to those used for human whole-breast 3D PA imaging [39], could be developed to cover the GI tract in human neonates.

## CRediT authorship contribution statement

**Tingting Huang:** Writing – review & editing, Writing – original draft, Visualization, Validation, Software, Resources, Methodology, Data curation, Conceptualization. **Li Li:** Resources, Methodology. **Yu Sun:** Resources, Methodology. **Yibing Wang:** Visualization, Resources, Methodology. **Xinlin Hou:** Supervision, Funding acquisition. **Changhui Li:** Writing – original draft, Visualization, Supervision.

## Declaration of competing interest

The authors declare that they have no known competing financial interests or personal relationships that could have appeared to influence the work reported in this paper.

## Data Availability

Data will be made available on request.

## Appendix A Supplementary material

Video S7

MMC S1

## References

[1] Johnson L.R. (2006).

[2] Penninck D., d’Anjou M.-A. (2015). Atlas of Small Animal Ultrasonography.

[3] Healy D.B., Ryan C.A., Ross R.P., Stanton C., Dempsey E.M. (2022). Clinical implications of preterm infant gut microbiome development. Nat. Microbiol..

[4] Groer M.W., Luciano A.A., Dishaw L.J., Ashmeade T.L., Miller E., Gilbert J.A. (2014). Development of the preterm infant gut microbiome: A research priority. Microbiome.

[5] Hull M.A., Fisher J.G., Gutierrez I.M., Jones B.A., Kang K.H., Kenny M., Zurakowski D., Modi B.P., Horbar J.D., Jaksic T. (2014). Mortality and management of surgical necrotizing enterocolitis in very low birth weight neonates: A prospective cohort study. J. Am. Coll. Surg..

[6] Kim J.H., Sampath V., Canvasser J. (2020). Challenges in diagnosing necrotizing enterocolitis. Pediatr. Res..

[7] Niño D.F., Sodhi C.P., Hackam D.J. (2016). Necrotizing enterocolitis: New insights into pathogenesis and mechanisms. Nat. Rev. Gastroenterol. Hepatol..

[8] Alsaied A., Islam N., Thalib L. (2020). Global incidence of necrotizing enterocolitis: A systematic review and meta-analysis. BMC Pediatr..

[9] Black C.J., Drossman D.A., Talley N.J., Ruddy J., Ford A.C. (2020). Functional gastrointestinal disorders: Advances in understanding and management. Lancet.

[10] Laghi A., Rengo M., Graser A., Iafrate F. (2013). Current status on performance of CT colonography and clinical indications. Eur. J. Radiol..

[11] Fidler J.L., Guimaraes L., Einstein D.M. (2009). MR imaging of the small bowel. Radiographics.

[12] Steinsvik E.K., Hatlebakk J.G., Hausken T., Nylund K., Gilja O.H. (2021). Ultrasound imaging for assessing functions of the GI tract. Physiol. Meas..

[13] Haase A., Gregersen T., Schlageter V., Scott M., Demierre M., Kucera P., Dahlerup J., Krogh K. (2014). Pilot study trialling a new ambulatory method for the clinical assessment of regional gastrointestinal transit using multiple electromagnetic capsules. Neurogastroenterol Motility.

[14] Beard P. (2011). Biomedical photoacoustic imaging. Interface Focus..

[15] Lin L., Wang L.V. (2022). The emerging role of photoacoustic imaging in clinical oncology. Nat. Rev. Clin. Oncol..

[16] Li C., Wang L.V. (2009). Photoacoustic tomography and sensing in biomedicine. Phys. Med. Biol..

[17] Wang L.V., Hu S. (2012). Photoacoustic tomography: in vivo imaging from organelles to organs. Science.

[18] Bézière N., Ntziachristos V. (2011). Optoacoustic imaging: an emerging modality for the gastrointestinal tract. Gastroenterology.

[19] Sun Y., Wang Y., Li W., Li C. (2024). Real-time dual-modal photoacoustic and fluorescence small animal imaging. Photoacoustics.

[20] Park J., Choi S., Knieling F., Clingman B., Bohndiek S., Wang L.V., Kim C. (2025). Clinical translation of photoacoustic imaging. Nat. Rev. Bioeng..

[21] Kim J., Lee J., Choi S., Lee H., Yang J., Jeon H., Sung M., Kim W.J., Kim C. (2024). 3D multiparametric photoacoustic computed tomography of primary and metastatic tumors in living mice. ACS Nano.

[22] Yang J., Choi S., Kim J., Lee J., Kim W.J., Kim C. (2025). Multiplane spectroscopic whole-body photoacoustic computed tomography of small animals in vivo. Laser Photonics Rev..

[23] Choi S., Kim J., Jeon H., Lee Y., Lim G., Ju W.M., Kim K., Lee D.S., Lee Y.S., Lee J.S., Cheon G.J., Choi Y., Kim C. (2026). Photoacoustic computed tomography monitors cerebrospinal fluid dynamics and glymphatic function. Nat. Commun..

[24] van der Heide M., Hulscher J.B., Bos A.F., Kooi E.M. (2021). Near-infrared spectroscopy as a diagnostic tool for necrotizing enterocolitis in preterm infants. Pediatr. Res..

[25] Weis J.A., Rauh J.L., Ellison M.A., Cruz-Diaz N., Yamaleyeva L.M., Welch C.D., Zeller K.A., Weis V.G. (2023).

[26] Paulus L.-P., Wagner A.L., Buehler A., Raming R., Jüngert J., Simon D., Tascilar K., Schnell A., Günther J., Rother U. (2023). Multispectral optoacoustic tomography of the human intestine–temporal precision and the influence of postprandial gastrointestinal blood flow. Photoacoustics.

[27] Zhou J., Ou M., Yuan B., Yan B., Wang X., Qiao S., Huang Y., Feng L., Huang L., Luo Y. (2025). Dual-modality ultrasound/photoacoustic tomography for mapping tissue oxygen saturation distribution in intestinal strangulation. Photoacoustics.

[28] Paulus L.-P., Buehler A., Wagner A.L., Raming R., Jüngert J., Simon D., Tascilar K., Schnell A., Rother U., Eckstein M. (2023). Contrast-enhanced multispectral optoacoustic tomography for functional assessment of the gastrointestinal tract. Adv. Sci..

[29] Zhang Y., Jeon M., Rich L.J., Hong H., Geng J., Zhang Y., Shi S., Barnhart T.E., Alexandridis P., Huizinga J.D. (2014). Non-invasive multimodal functional imaging of the intestine with frozen micellar naphthalocyanines. Nature Nanotechnology.

[30] Huang W., Chen R., Peng Y., Duan F., Huang Y., Guo W., Chen X., Nie L. (2019). In vivo quantitative photoacoustic diagnosis of gastric and intestinal dysfunctions with a broad pH-responsive sensor. ACS Nano.

[31] Bhutiani N., Samykutty A., McMasters K.M., Egilmez N.K., McNally L.R. (2019). In vivo tracking of orally-administered particles within the gastrointestinal tract of murine models using multispectral optoacoustic tomography. Photoacoustics.

[32] Swinbank R., James Purser R. (2006). Fibonacci grids: A novel approach to global modelling. Q. J. R. Meteorol. Soc.: A J. Atmos. Sci. Appl. Meteorol. Phys. Ocean..

[33] (2022).

[34] Wang Y., Li C. (2024). Comprehensive framework of GPU-accelerated image reconstruction for photoacoustic computed tomography. J. Biomed. Opt..

[35] Hall J.E., Hall M.E. (2026).

[36] Huizinga J.D., Chen J.-H., Fang Zhu Y., Pawelka A., McGinn R.J., Bardakjian B.L., Parsons S.P., Kunze W.A., Wu R.Y., Bercik P. (2014). The origin of segmentation motor activity in the intestine. Nat. Commun..

[37] Bashashati M., Andrews C.N., Chen J.D., McCallum R.W., Koch K.L. (2026). Gastric electrophysiology. Expert. Rev. Gastroenterol. Hepatol..

[38] Martínez-Ruiz S., Olivo-Martínez Y., Cordero C., Rodríguez-Lagunas M.J., Pérez-Cano F.J., Badia J., Baldoma L. (2024). Microbiota-derived extracellular vesicles as a postbiotic strategy to alleviate diarrhea and enhance immunity in rotavirus-infected neonatal rats. Int. J. Mol. Sci..

[39] Dantuma M., Lucka F., Kruitwagen S., Javaherian A., Alink L., van Meerdervoort R.P., Nanninga M., Root T., De Santi B., Budisky J. (2023). https://arxiv.org/abs/2308.06754.

