# Supplementary material for: Quantitative assessment of gastrointestinal motility in neonatal rats by real-time 3D photoacoustic imaging: A feasibility study
Source: Photoacoustics. 2026 Jul 13;51:100853. doi: 10.1016/j.pacs.2026.100853 (PMC13382320; doi:10.1016/j.pacs.2026.100853)
Supplement: MMC S1 [file mmc8.pdf]

Supplementary Material

Quantitative Assessment of Gastrointestinal Motility in Neonatal Rats by Real-time 3D Photoacoustic Imaging: A Feasibility Study

Supplementary Figures

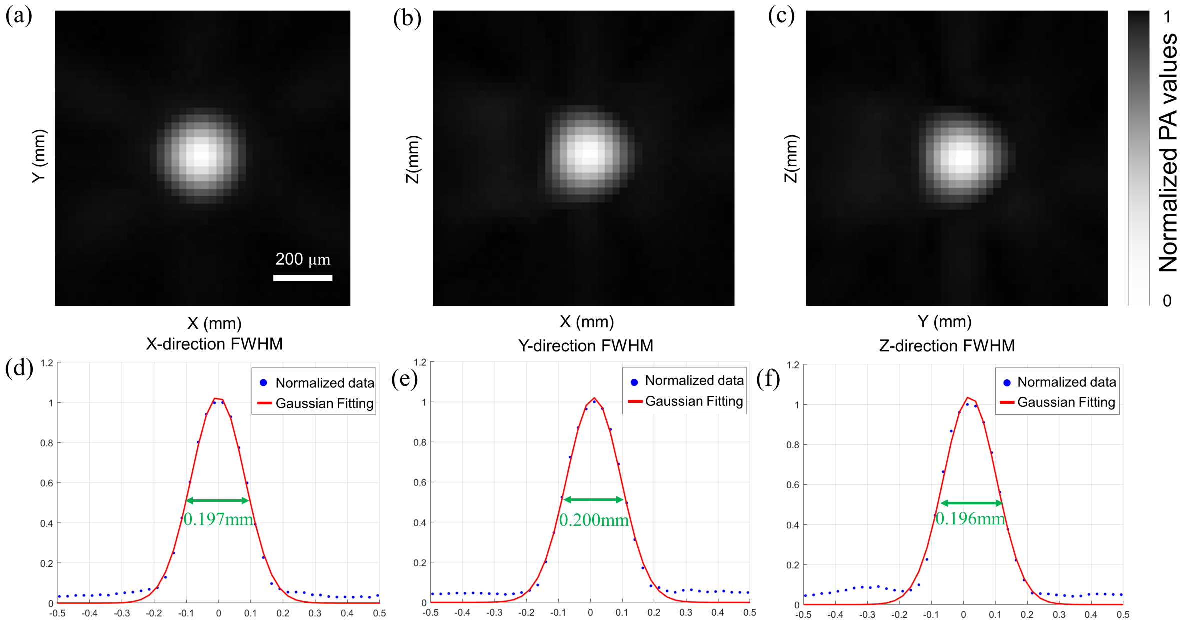

Supplementary Fig. S1: Spatial-resolution characterization using a point-like absorber. (a–c) Orthogonal maximum-intensity projections of the reconstructed point-like target in the XY, XZ, and YZ planes, respectively. The grayscale color bar represents normalized PA values, and the scale bar is 500 μm. (d–f) Normalized intensity profiles along the X, Y, and Z directions extracted through the signal maximum. Blue dots represent normalized experimental data, and red curves indicate Gaussian fitting results. The measured full widths at half maximum were 0.197 mm, 0.200 mm, and 0.196 mm along the X, Y, and Z directions, respectively.

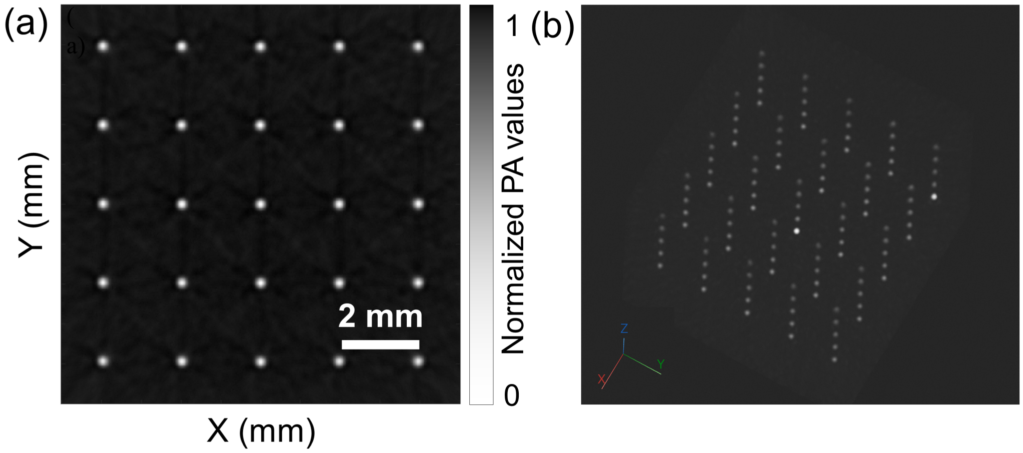

Supplementary Fig. S2: Spatial-position calibration and resolving capability evaluated with multiple point sources. (a) PA reconstruction cross-section of the multiple-point-source sample in the XY plane. (b) 3D PA reconstruction of 125 point sources scanned with a 2 mm step size around the center of the imaging field. The grayscale values represent normalized PA signal intensity. Scale bar: 2 mm.

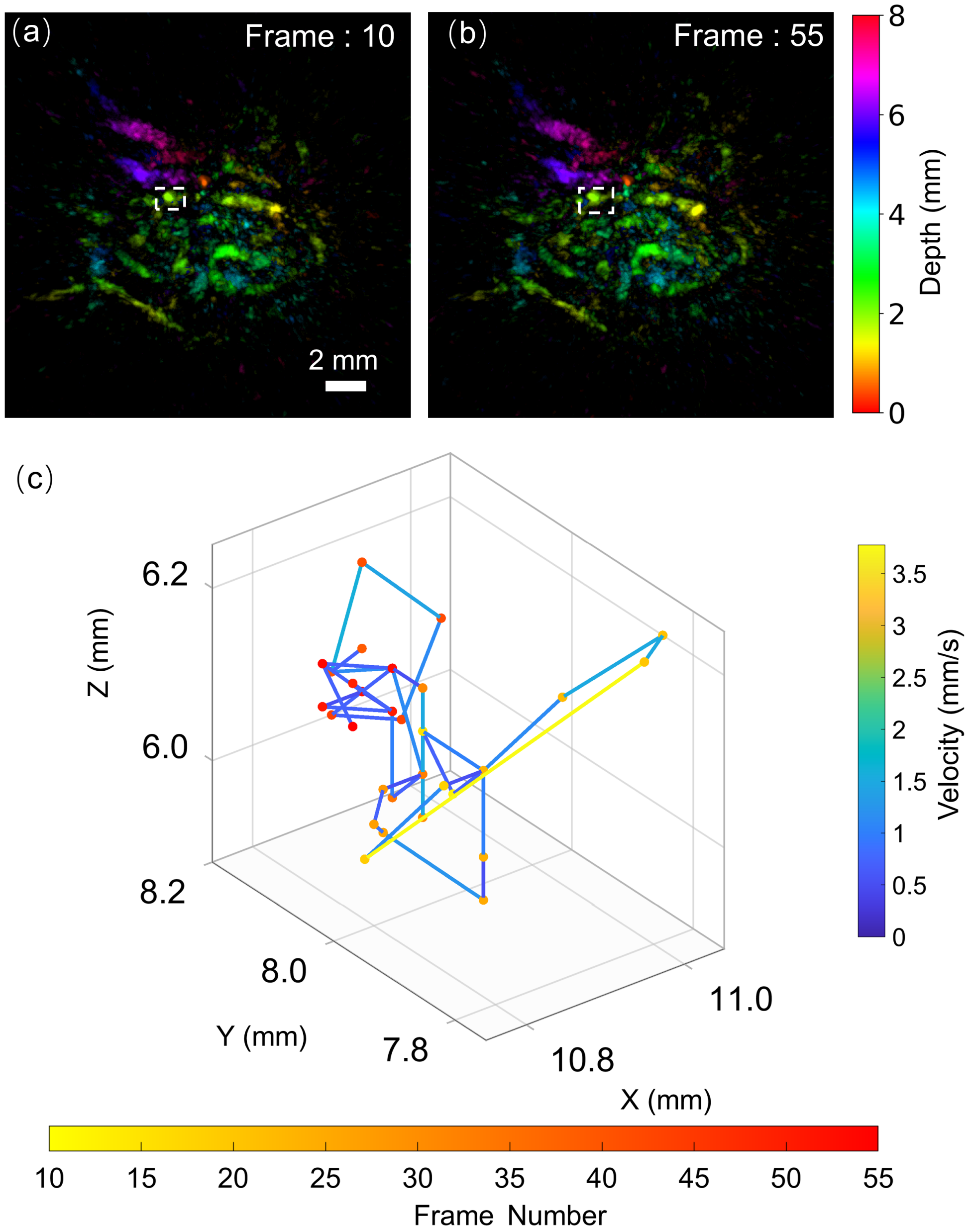

Supplementary Fig. S3: Contrast-free intraluminal cluster tracking analysis. (a) Maximum amplitude projection (MAP) image of the 3D PA sequence at frame 10. The white dashed box indicates the tracked cluster used for analysis. (b) MAP image at frame 55, with the white dashed box indicating the tracked cluster. (c) Visualization of the 3D trajectory of the tracked point from frames 10 to 55. Line segments are colored by speed in mm/s, and scatter points are colored by frame number. The tracked endogenous cluster moved within a limited local region and showed lower speed than the exogenous contrast-agent clusters analyzed in the main text.

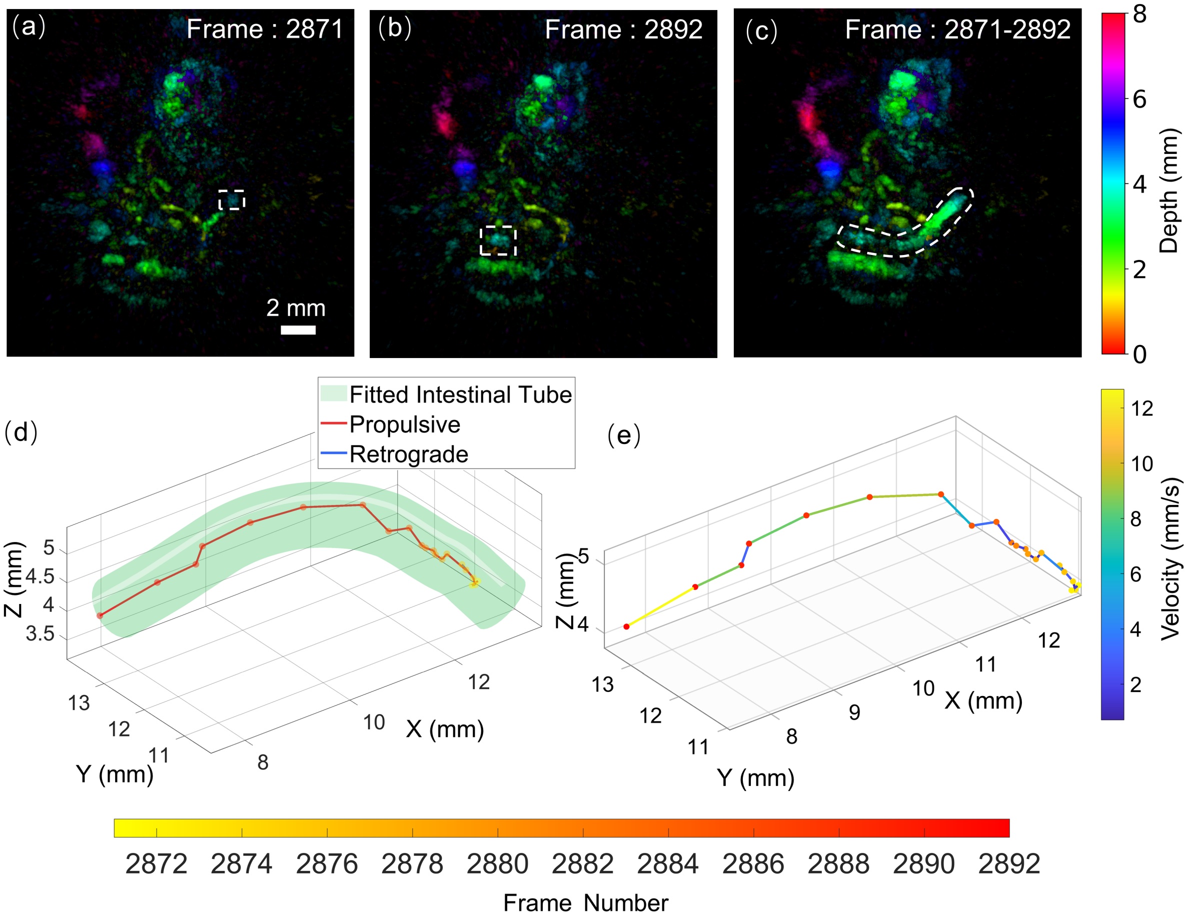

Supplementary Fig. S4: Repeatability analysis of contrast-agent tracking and peristaltic-pattern classification at a different time point within the same intestinal lumen segment. (a) MAP image of the 3D PA sequence at frame 2871. The white dashed box indicates the tracked cluster used for analysis. (b) MAP image at frame 2892. (c) tMVP image generated from frames 2871–2892, with the white dashed outline marking the overall migration pathway of the cluster. (d) Classification of intestinal motion based on local motion direction. The semitransparent green structure represents a virtual intestinal segment; red markers indicate propulsive motion, and blue markers indicate retrograde motion. (e) Visualization of the 3D trajectory of the tracked point. Line segments are colored by speed in mm/s, and scatter points are colored by frame number.

Supplementary Videos

List and description of supplementary videos.

Video name	Content
Supplementary Video 1	Three-dimensional animation of the contrast-free PA reconstruction of the neonatal rat intestine.
Supplementary Video 2	Dynamic intestinal peristaltic process over a 60 s interval from 180 s to 240 s.
Supplementary Video 3	Continuous dynamic PA imaging of the gastrointestinal tract acquired 10 min after oral gavage of India ink.
Supplementary Video 4	Three-dimensional animation of the depth-encoded tMVP reconstruction generated from all 3,600 frames acquired 10 min after gavage.
Supplementary Video 5	Dynamic transportation of a tracked intraluminal contrast-agent cluster driven by intestinal peristalsis.
Supplementary Video 6	Three-dimensional dynamic animation of intestinal imaging 24 h after contrast-agent gavage.
Supplementary Video 7	Long-duration intestinal recording 24 h after contrast-agent gavage.
